# The relevance of climate change and sustainability in nursing education: a cross-sectional study of students’ perspectives

**DOI:** 10.1186/s12912-025-03285-5

**Published:** 2025-07-03

**Authors:** Jennie Aronsson, Marie Elf, Paul Warwick, Riccardo LoMartire, Anna Anåker

**Affiliations:** 1 Faculty of Health, School of Nursing and Midwifery, University of Plymouth, Plymouth, Devon, UK; 2 School of Health and Welfare, Dalarna University, Falun, Sweden; 3 Plymouth Institute of Education, School of Society and Culture, University of Plymouth, Plymouth, Devon, UK; 4 Centre for Clinical Research Dalarna, Uppsala University, Falun, Sweden

**Keywords:** Sustainability, Climate change, Nursing education, Students’ perspectives, Spiral curriculum

## Abstract

**Background:**

As climate change continues to challenge global health, nursing education must evolve to prepare future nurses for the impacts on care delivery and promote sustainable practices within the healthcare system which is itself a contributor to the climate crisis through emissions and waste. In this study we aimed to i) explore undergraduate nursing students’ attitudes towards and awareness of climate change and sustainability issues and its inclusion in nursing education at a Swedish university, and ii) explore differences in awareness and attitudes across student groups in different semesters.

**Methods:**

In this cross-sectional descriptive research study, nursing students from six groups (semester 1,2,3,4,5,6) of a three-year BSc Nursing programme were asked to complete the Sustainability Attitudes in Nursing Survey (SANS_2) questionnaire. Descriptive statistics were used to present mean scores for each item, and the Kruskal-Wallis test was used to identify differences in responses between different student groups. The Strengthening the Reporting of Observational Studies in Epidemiology (STROBE) statement has been used to report this research.

**Results:**

A total of 211 (32%) students completed the questionnaires. The findings showed that nursing students across the six semesters had relatively good awareness of, and positive attitudes towards, sustainability and climate change (no mean rating below 4 on a scale 1–7). Statistically significant differences between student groups were found in four out of nine items of the SANS_2 questionnaire, with students in their second semester consistently providing the highest ratings (p < 0.05). Notably, the lowest ratings across these four items were provided by students in their last year of the programme.

**Conclusions:**

This study found that whilst students initially felt equipped to address sustainability issues and climate change in practice, this confidence appears to diminish by the programme’s end, raising concerns about the preparedness of the future workforce. This underscores the urgency and importance of continuous reinforcement of sustainability concepts in nursing education, aligning with a spiral curriculum approach which advocates for reiterating themes throughout the curriculum to deepen understanding.

**Clinical trial number:**

Not applicable.

## Background

Climate change is a growing threat to global health, placing new demands on healthcare systems and professionals, particularly nurses, who play a critical role in addressing its impacts and promoting sustainable practices [1, 2]. Climate change refers to long-term shifts in global or regional climate patterns, primarily driven by human activities such as burning fossil fuels, deforestation, and industrial emissions, which result in rising temperatures, extreme weather events, and significant health impacts [3]. Whilst integrating sustainability and climate-related topics into nursing curricula is essential for preparing graduates to tackle these challenges, gaps remain in effectively embedding and reinforcing these concepts throughout educational programs [4]. Sustainability is the practice of meeting current needs without compromising the ability of future generations to meet their own needs, encompassing environmental, social, and economic dimensions [5]. In healthcare, this involves reducing resource use, emissions, and waste whilst promoting equitable and effective care [6].

Anthropogenic climate change is intensifying extreme weather events, threatening human health, and reversing the global health gains over the past 50 years [7]. Today there are worries that climate changes push beyond ecological boundaries or critical thresholds in the Earth system, known as planetary boundaries, creating unsafe living conditions due to pollution and biodiversity loss [8]. Climate change disproportionately impact vulnerable populations, exacerbating health inequalities and placing those least responsible for climate change at greatest risk [9]. Nurses, as the largest health profession worldwide, play a crucial role in addressing these challenges through education and proactive engagement in climate change mitigation and adaptation strategies [10]. The International Council of Nursing (ICN) and UNESCO emphasize the need for nurses to serve as planetary citizens, advocating for sustainable development and adapting to climate impacts [11]. At the same time, the healthcare sector is a major contributor to carbon emissions, mainly due to energy consumption, medical waste and resource-intensive practices. This highlights the urgent need for sustainable healthcare practices that reduce environmental impact whilst addressing the growing health challenges of climate change [1].

Nursing education plays a crucial role in equipping future nurses with the knowledge, attitudes, and skills to address climate change’s impact on healthcare delivery. By integrating sustainability and climate-related topics into curricula, nursing programs can prepare graduates to not only adapt to these challenges but also promote sustainable practices within healthcare systems, helping to mitigate emissions and waste contributing to the climate crisis [11]. However, current nursing educational programmes vary in how they address sustainability [12], highlighting the need for higher education to evaluate and enrich curricula with comprehensive training on climate science, sustainable healthcare practices, and adaptation strategies. This can enhance student awareness and knowledge and also bridge the gap between theoretical learning and practical application in clinical settings [13].

Integrating climate change and sustainability into nursing curricula is increasingly recognized as essential to prepare nurses for emerging global health challenges. Quinn et al [14] advocate for embedding climate change content within existing nursing programs to equip future nursing leaders with the knowledge and skills necessary to address policy and practice implications. Neal-Boylan et al [15] highlight significant gaps in nursing education, emphasizing the need for comprehensive and strategic inclusion of climate change topics to address its health impacts. Topics like resource use, health promotion, and the environmental impact of healthcare delivery are crucial to foster environmental sustainability competencies in nursing students [16]. Furthermore, Oerther and Rosa [17] argue that nurses need an understanding about the Sustainable Development Goals (SDGs), in order to address health care inequalities and preventive care. Nursing students therefore need to study the interdependence of all SDGs, the social and financial determinants of health, and the impact of climate change on health and wellbeing in theory, as well as be exposed to these questions and dilemmas in clinical practice. Shaw et al [1] propose practical strategies to enact this vision, such as case-based learning, resource-efficient clinical practices, and interdisciplinary projects.

Integrating climate-related content and innovative pedagogical strategies can prepare graduates to address climate challenges and advocate for sustainable practices within healthcare systems, which contribute to the climate crisis through emissions and waste. However, a gap remains in understanding the extent to which these topics are effectively integrated into current nursing education. Thus, in this study we aimed to i) explore undergraduate nursing students’ attitudes towards and awareness of climate change and sustainability issues and its inclusion in nursing education at a Swedish university, and ii) explore differences in awareness and attitudes across student groups in different semesters.

## Material and methods

### Study design

A cross-sectional descriptive research design was employed. The Strengthening the Reporting of Observational Studies in Epidemiology (STROBE) statement has been used to report this research [18].

### Setting

The study was undertaken at a university located in the central region of Sweden. This was selected based on the authors’ actively engaging with sustainability education within the nursing programme at this university. Furthermore, it was expected that the findings from the study would contribute to the development of educational strategies and teaching methods in sustainability at this institution, as well as elsewhere.

The BSc nursing programme at the chosen university comprises of six semesters over three years, with sustainability education being mainly included within a module delivered in semester 2 (end of the first year on the programme), but also touched upon very briefly in semester 5 and 6 (year 3) (Table 1).

**Table 1:** Overview of sustainability in the curriculum

Year 1		Year 2		Year 3	
*Semester 1 (S1)*	*Semester 2 (S2)*	*Semester 3 (S3)*	*Semester 4 (S4)*	*Semester 5 (S5)*	*Semester 6 (S6)*
None	Describe how drugs affect the environment and discuss how management can become more sustainable from an environmental perspective. Describe the consequences of current climate change in relation to the terms ecological, economic, and social sustainability as a component of sustainable development. Demonstrate an understanding of global sustainability goals and national public health goals from the perspectives of the individual, family, society, using a multicultural lens.	None	None	Reflect on the management of residual waste and supplies in relation to cost effectiveness, the environment, and a sustainable society.	Reflect on the management of consumables and drugs in relation to cost-effectiveness, the environment and a sustainable society.

### Participants recruitment

Nursing students from six groups (semester 1–6) of the three-year BSc Nursing programme were approached via an email with a link to the electronic survey in November 2023. Reminders were sent out on two occasions in December 2023 and January 2024. All students who voluntarily agreed to participate were included. Incomplete or incorrectly filled out questionnaires were excluded.

### Data collection

The SANS_2 questionnaire was used to collect data on nursing students’ attitudes towards and awareness of climate change and sustainability and the relevance of these concepts to nursing and nursing education. SANS_2 is a validated instrument, which has been translated into several European languages [19]. The instrument was translated to Swedish by the research team, where the first author is bilingual. An iterative approach was used, whereby each question was read out in both languages and discussed to ensure that the questionnaire would be user-friendly without losing meaning. The final version was translated back to English and reviewed by the original developer of the instrument (Professor Emerita Janet Richardson) to ensure conceptual accuracy. The resulting instrument includes nine statements in Swedish, outlined in Table 2.

**Table 2:** SANS_2 questionnaire

1. Climate change is an important issue for nursing
2. Issues about climate change should be included in the nursing curriculum
3. Sustainability is an important issue for nursing
4. Sustainability should be included in the nursing curriculum
5. I apply sustainability principles at home
6. I apply sustainability principles during my clinical placements
7. During clinical placements, I have noticed unsustainable practice
8. I feel that it is hard for me to challenge unsustainable practice within clinical placements
9. I have been able to apply my knowledge about sustainability during clinical placements

The students were asked to respond to the statements on a Likert scale from 1–7 (strongly disagree – strongly agree). Students in semester 1 had not yet had any clinical placements, and therefore they only responded to items 1–5. Items are valued separately, with no summa score, and in this study, the attitudes and awareness of students across different semesters were compared. Additionally, demographic data was collected.

### Data analysis

IBM SPSS Statistics Version 28.0.1.1 was used to generate descriptive statistics and rankFD v0.1.1 in R v4.3.3 for the hypothesis tests.

Individual item scores were compared between all semesters simultaneously using the unweighted Kruskal-Wallis test based on pseudo-ranks, which is recommended for comparisons of groups with unequal sample sizes [20]. For items where the null hypothesis was rejected (i.e. where we found support for a non-equivalence between semesters), we also compared the semesters pairwise, using multiple contrast tests to asymptotically maintain the familywise error rate by item [21].

### Ethical consideration

An ethics committee at the university where the research was conducted granted ethical approval for the study (DNR 4.2–2016/556). All students received information regarding the study and signed the consent form prior to participating in the study. Participation was voluntary, and students had the right to withdraw without adverse effects on their academic standing. The confidentiality of personal data was maintained: student details were not recorded on the questionnaire, and teachers were not aware of specific student responses. The data collected were used for specific research purposes and kept in the custody of the researchers.

## Results

The questionnaires were sent to a total of 669 students in the BSc Nursing programme, and a total of 211 (32%) complete questionnaires were returned.

### Demographic data

The respondents included students from all semesters, with an even distribution between semesters, apart from semester 4 and 6 which had smaller samples. Out of the total sample of 211 respondents, 169 were female (80%). Most respondents fell within the 19–25 age range (25%) or the 26–30 age range (25%). In addition, 69% reported having prior healthcare experience. This trend was relatively consistent across the semesters. The demographic profile grouped by semester (S1 – S6) is outlined in Table 3.

**Table 3:** Demographic profile

Semester n (response rate %)	S1 61 (51)	S2 36 (34)	S3 37 (37)	S4 18 (13)	S5 41 (32)	S6 18 (25)
Gender n (%)						
Female Male	45 (74) 16 (26)	30 (83) 6 (17)	32 (86) 5 (14)	14 (78) 4 (22)	35 (85) 6 (15)	13 (72) 5 (28)
Age n (%)						
19–25 26-30 31–35 36-40 >41	22 (36) 14 (23) 7 (11) 7 (11) 11 (18)	8 (22) 9 (25) 6 (17) 4 (11) 9 (25)	8 (22) 8 (22) 7 (19) 8 (22) 6 (16)	3 (17) 3 (17) 4 (22) 2 (11) 6 (33)	11 (27) 11 (27) 7 (17) 2 (5) 10 (24)	1 (6) 7 (39) 5 (28) 3 (17) 2 (11)
Prior healthcare experience n (%)						
Yes No	44 (72) 17 (28)	30 (83) 6 (17)	26 (70) 11 (30)	11 (61) 7 (39)	21 (51) 20 (49)	13 (72) 5 (28)
Sustainability education in last 3 months (recall) n (%)						
Yes No	11 (18) 50 (82)	28 (78) 8 (22)	21 (57) 16 (43)	2 (11) 16 (89)	3 (7) 38 (93)	5 (28) 13 (72)

### Student attitudes towards and awareness of climate change and sustainability

Students generally acknowledged climate change as an important issue for nursing, with the highest levels of agreement observed in S2 (M = 6.1), followed by a decline in later semesters, particularly in S6 (M = 4.8). Similarly, students’ agreement on the inclusion of climate change in the curriculum followed a comparable pattern, peaking in S2 (M = 5.8) and declining in S6 (M = 4.6).

Regarding sustainability as an important issue in nursing, responses remained consistently high across all semesters, ranging from 5.7 in S1 and S5, to 6.1 in S2 and S4, indicating that students largely agree on the relevance of sustainability to their profession. However, when considering the inclusion of sustainability within the curriculum, more variation was observed, with the highest support in S2 (M = 6.0) and the lowest in S1 and S4 (M = 5.3).

Students reported that they applied sustainability principles at home (5.2–5.8) and, to a moderate extent, in clinical settings (4.8–5.9). Students’ ability to notice unsustainable practices in clinical placements followed a similar trend, with scores ranging from 4.4 in S6 to 5.3 in S5, suggesting awareness but not necessarily action. Similarly, students reported challenges in confronting unsustainable practices (4.1–5.1), indicating a need for more empowerment and training.

Table 4 presents the results from nursing students across the six semesters (S1–S6) regarding their perceptions of climate change and sustainability within their education and clinical practice.

**Table 4:** SANS_2 findings

Item	S1 Mean (SD)	S2 Mean (SD)	S3 Mean (SD)	S4 Mean (SD)	S5 Mean (SD)	S6 Mean (SD)
1. Climate change is an important issue for nursing	5.3 (1.5)	6.1 (1.5)	5.6 (1.6)	5.4 (1.8)	5.1 (1.5)	4.8 (1.9)
2. Issues about climate change should be included in the nursing curriculum	4.6 (1.8)	5.8 (1.8)	5.7 (1.9)	4.8 (2.3)	5.2 (1.8)	4.6 (2.3)
3. Sustainability is an important issue for nursing	5.7 (1.4)	6.1 (1.5)	6.0 (1.4)	6.1 (1.4)	5.7 (1.4)	5.9 (1.5)
4. Sustainability should be included in the nursing curriculum	5.3 (1.6)	6.0 (1.5)	5.8 (1.6)	5.3 (2.1)	5.7 (1.7)	5.7 (1.7)
5. I apply sustainability principles at home	5.4 (1.4)	5.8 (1.4)	5.4 (1.4)	5.3 (1.7)	5.6 (1.2)	5.2 (1.3)
6. I apply sustainability principles during my clinical placements	N/A	5.9 (1.6)	5.7 (1.7)	5.2 (1.7)	4.8 (1.6)	5.1 (1.4)
7. During clinical placements, I have noticed unsustainable practice	N/A	5.0 (1.8)	4.7 (2.1)	4.7 (2.1)	5.3 (1.6)	4.4 (1.8)
8. I feel that it is hard for me to challenge unsustainable practice within clinical placements	N/A	4.1 (2.1)	4.3 (1.8)	4.8 (1.8)	5.1 (1.7)	4.7 (2.2)
9. I have been able to apply my knowledge about sustainability during clinical placements	N/A	5.4 (1.8)	5.1 (1.9)	4.8 (1.5)	4.3 (1.7)	4.1 (1.3)

### Post-hoc pairwise comparison

The comparative analysis suggested varying perceptions and engagement with sustainability across semesters, particularly between S2, S5, and S1. More specifically, differences were found for the statements on climate change being important for nursing (S2 vs. S5, p < 0.05) and its inclusion in the curriculum (S2 vs. S1, S3 vs. S1, p < 0.05). While no statistically significant differences were observed for sustainability’s importance or home application (p > 0.05), significant differences emerged in applying sustainability during clinical placements (S2 vs. S5, p < 0.05) and applying sustainability knowledge (S2 vs. S6, p < 0.05).

Table 5 shows the comparative analysis between semesters, for each item in the questionnaire. The first column shows if there was a significant difference (p-value < 0.05 shows that there is a statistical difference in the group). In cases where there is a difference, the second column shows between which semesters (S1 – S6) this difference exists.

**Table 5:** Comparison between semesters

Item	Statistical difference between any semesters p-value	The semesters where the difference exists (the first one presented has the higher SANS_2 score)
1. Climate change is an important issue for nursing	0.016	S2 vs S5
2. Issues about climate change should be included in the nursing curriculum	0.0054	S2 vs S1 and S3 vs S1
3. Sustainability is an important issue for nursing	0.15	N/A
4. Sustainability should be included in the nursing curriculum	0.25	N/A
5. I apply sustainability principles at home	0.45	N/A
6. I apply sustainability principles during my clinical placements	0.0051	S2 vs S5
7. During clinical placements, I have noticed unsustainable practice	0.47	N/A
8. I feel that it is hard for me to challenge unsustainable practice within clinical placements	0.25	N/A
9. I have been able to apply my knowledge about sustainability during clinical placements	0.031	S2 vs S6

**Fig. 1:**
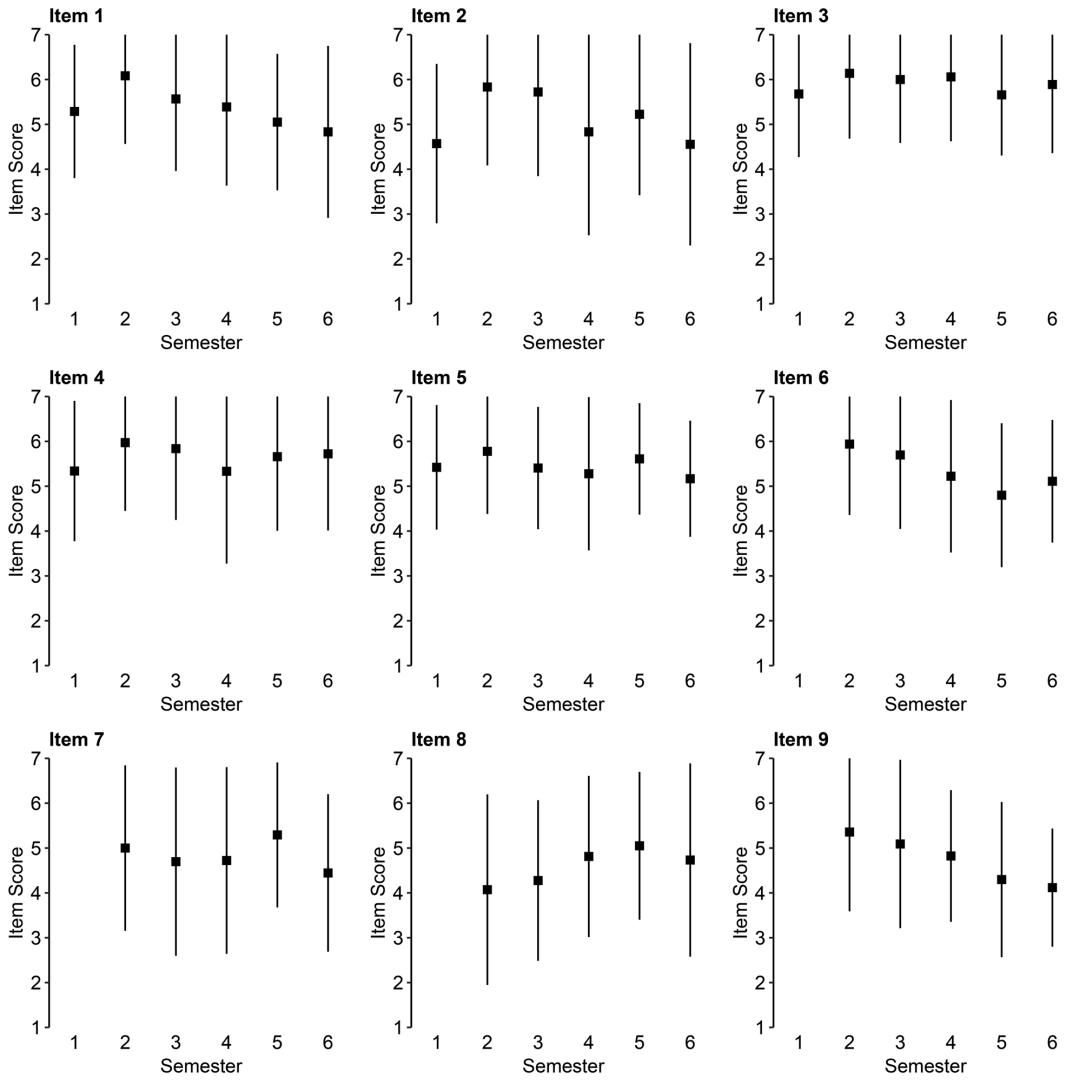
Changes across semester

Figure 1 provides a visualisation of changes across semester.

## Discussion

This study provides important information about nursing students’ views and understanding of sustainability and climate change during their education. The high number of respondents with healthcare experience makes these findings particularly relevant to professional practice and nursing education; as Richardson et al [22] point out, clinical experience is relevant in terms of building confidence and applying knowledge in practice. Students’ insights, based on their real-world experiences, can help develop future curricula and practices in nursing education.

The findings showed that students’ perceptions of the importance of climate change in nursing and the necessity of incorporating climate-related issues into the nursing curriculum peaked early on in their studies: students in semester 2 (S2) consistently provided higher scores than the other respondents. This could be due to the integration of in-depth sustainability education in their second semester, which covers the impacts of climate change on health, the SDGs, and sustainable practice in nursing. Previous research indicates a direct link between sustainability education and enhanced knowledge of climate change and sustainability issues, as well as positive attitudes towards their inclusion within nursing curricula [23–25]. This is supported by our findings that students in semester 1 (S1) rated that climate change should be included in the nursing curriculum as low, possibly due to not having received any sustainability education yet.

Notably, the lowest rating for climate change and sustainability as important issues for nursing occurred late in the programme. Although the students had some education on sustainable healthcare in semesters 5 and 6, this does not explicitly cover climate change and the SDGs. The lower scores could be linked to the time that had lapsed from taking the S2 course but might also be related to students gaining more insight into the complexities of nursing practice, which may shift focus away from sustainability principles. This was found in the study by Anåker et al [26], wherein some nursing students struggled to see the relevance of sustainability issues in a busy nursing context where the priority is to save lives. The decline in awareness and positive attitudes within our study highlights the challenges of integrating sustainability into a curriculum dominated by clinical skills and competencies. It might also be that the pedagogical approaches employed in S2 were more effective in promoting student engagement and therefore resulted in deeper learning. A systematic review by Lopez-Medina et al [16] found that sustainability educational activities that are flexible, diverse and involve reflection are more likely to lead to increased knowledge and positive attitudes in nursing students.

The most concerning findings are about sustainability in clinical practice, with the lowest scores reported for item 6 (I apply sustainability in my clinical placements) in semester 5, and for item 9 (I have been able to apply my knowledge of sustainability during clinical placements) in semester 6. This is confusing given that students received targeted sustainability education during these periods, focusing on clinical practice aspects such as procurement and waste management. However, it is crucial to recognize that students’ attitudes towards climate change are not solely shaped by sustainability education, but also by the prevailing societal discourse. The broader social atmosphere, including public debates and media coverage of environmental issues, significantly influences students’ perceptions and engagement with climate change [27]. Yet this does not fully explain the results. The declining levels of awareness and positive attitudes indicate a significant gap in the effectiveness of sustainability education, particularly in its clinical application. Aronsson et al [13] demonstrated that the ability to apply theory related to climate change and sustainability in practice is linked to experienced confidence during placements. Whilst we did not measure the students’ self-confidence, it is possible that our students did not feel sufficiently confident to raise sustainability issues during their placement. If this is the case, this indicates a need to empower students to be change agents in practice, as supported by Power [28], as well as consider the specific nature and dominant culture of the placements themselves, the opportunities provided and the resistance or inertia of each care context to apply sustainability principles. This mirrors findings from the study by Aronsson et al [29], which found that students often lacked confidence to challenge unsustainable practices during clinical placements due to resistance in the clinical environment.

For the items ‘Sustainability is an important issue for nursing,’ ‘Sustainability should be included in the nursing curriculum,’ ‘I apply sustainability principles at home,’ and ‘During clinical placements, I have noticed unsustainable practices,’ some variation in ratings was observed between semesters. Whilst these differences were not statistically significant, the overall trend for these items were consistent with those showing significant differences. It is important to note that all items received relatively high ratings (no mean below 4), which suggests that the students generally have a good understanding and positive opinions about sustainability. Although this is positive, educators should focus on deepening their understanding and encouraging critical thinking about the complex issues related to sustainability in healthcare, therefore challenging students to enter a theoretical space wherein they are exposed to new ways of understanding or interpreting a topic. This is what Meyer and Land [30] call troublesome knowledge, a transformative approach to teaching and learning.

A key takeaway from this study is the critical need for sustainability to be a consistent thread throughout the nursing curriculum rather than being addressed at a single point in a few modules. There is growing evidence suggesting that sustainability education within nursing curricula improves awareness, attitudes, and skills related to climate change and sustainability. For example, Álvarez-García et al [23] found that students who participated in a 90 minute taught case-based learning session gained significant environmental knowledge, skills and attitudes; Grose et al [31] explored interdisciplinary pedagogies for sustainability education and found that students could see the link of their learning to practice settings; and Linton et al [32] evaluated a 4-week sustainability in nursing module and found that students’ attitudes about sustainability in practice significantly improved. However, knowledge is perishable, and to establish ‘habits of mind,’ it is crucial that topics and concepts are revisited and built upon over time [33]. This underscores the urgency and importance of continuous reinforcement of sustainability concepts in nursing education, aligning with the spiral curriculum approach suggested by Light [34], which advocates for reiterating themes throughout the curriculum to deepen understanding. Consistent integration of sustainability, tailored to different stages of the curriculum, could improve students’ awareness and attitudes towards sustainability and climate change and the relevance of these concepts to their future professional roles, potentially enhancing their impact in clinical settings.

The findings in our study support the need for sustainability education to be integrated into core disciplinary curricula for lasting learning, consistent with global sustainability education research across other professional subject areas in Higher Education, which suggest that a single sustainability course is unlikely to lead to transformative learning and systemic change [35]. Given the rapid changes in healthcare systems and the increasing need for leadership in sustainable practices, integrating sustainability education throughout nursing programmes becomes crucial [2]. Shaw et al [1] advocate for eco-ethical leadership, emphasizing values like sustainability, collaboration, and justice aimed at advocacy and action, which is particularly relevant for inclusion in the latter stages of nursing programmes to prepare students for leadership roles as registered nurses. Roden et al [10] propose that this role should include leadership for planetary health in nursing practice, policy and research, and conclude that education is central to this. A collaborative approach between academic staff and clinical educators would facilitate development of sustainability education in nursing curricula; yet there is limited evidence on educators’ (both academic and clinical) perspectives and ideas around sustainability education [12]. Co-design of teaching material would be further improved by including students, in order to ensure that content and pedagogy meet students’ needs and expectations.

### Limitations

Our study is limited by its small sample size – in particular, there were only 18 students participating from semester 4 and semester 6 respectively. Whilst we accounted for the different sample sizes across groups in our comparisons, the limited number of participants in some semesters combined with the low response rate at 32%, makes our comparison susceptible to bias, which may complicate the generalisability of our results. However, there is no reason to believe that non-response would differ between semesters.

As this study used surveys, the insights could be enhanced by conducting interviews to provide a more comprehensive understanding of students’ attitudes and the long-term impact of sustainability education on nursing practice. It is also noteworthy that the study was cross-sectional and involved six different groups of students (semesters 1–6), making longitudinal conclusions difficult. It would be helpful to follow a cohort from the start of their nursing programme to the end to provide a deeper insight into the longitudinal nature of individual nursing students’ attitudes towards and awareness of climate change and sustainability change throughout their studies. It would also be helpful to compare this research across multiple higher education curricula.

An additional limitation of the study is the translated SANS_2 instrument has not undergone a formal validation by an expert committee. Although a bilingual expert performed a back-and-forth translation and the original developer reviewed it, some concerns about the instrument’s validity remains. However, the Swedish instrument has previously been used in a European study with comparable findings [36]. To enhance validity across different linguistic and pedagogical contexts, future research should conduct psychometric validation, including expert reviews and reliability testing.

## Conclusions

The results of this study suggest a correlation between sustainability education within the nursing programme, and positive attitudes towards the relevance of sustainability and climate change to nursing as well as the ability to apply theoretical knowledge in practice. Whilst the nursing students across all cohorts demonstrated good awareness and positive attitudes towards these concepts, the highest ratings were observed in semester 2, concordant with recent education on climate change and sustainable nursing practice. Conversely, lower ratings towards the end of the nursing programme suggest that these topics may not be explicitly reinforced at the latter part of the programme, particularly in relation to clinical application and the SDGs.

These results underscore the need for consistent integration of sustainability education throughout nursing programmes, tailored to different stages of the curriculum. Nursing educators should consider adopting a spiral curriculum approach, where sustainability and climate change are revisited and deepened across the programme. This could contribute to the development of apt nursing curricula that adequately prepare students as future healthcare providers and influential leaders equipped to address the dual challenge of promoting the health of the planet, as well as the health of the population inhabiting it.

## Data Availability

The datasets used and analysed during the current study are available from the corresponding author on reasonable request.
